# Prolonged overall survival in a child with multimetastatic retinoblastoma treated with anti-GD2 monoclonal antibody dinutuximab beta: a case report

**DOI:** 10.3389/fonc.2025.1665968

**Published:** 2025-10-17

**Authors:** Valentina Di Ruscio, Alessia Carboni, Giada Del Baldo, Maria Debora De Pasquale, Paola Valente, Angela Di Giannatale, Annalisa Serra, Rita De Vito, Angela Mastronuzzi, Concetta Quintarelli, Biagio De Angelis, Giuseppe Maria Milano, Ida Russo

**Affiliations:** ^1^ Department of Onco-Hematology, Cell and Gene Therapy, Bambino Gesù Children’s Hospital, Scientific Institute for Research, Hospitalization and Healthcare Istituto di Ricovero e Cura a Carattere Scientifico (IRCCS), Rome, Italy; ^2^ Neuroradiology Unit, “Bambino Gesù” Children’s Hospital Istituto di Ricovero e Cura a Carattere Scientifico (IRCCS), Rome, Italy; ^3^ Ophthalmology Unit, Bambino Gesù Istituto di Ricovero e Cura a Carattere Scientifico (IRCCS) Children’s Hospital, Rome, Italy; ^4^ Pathology Unit, Department of Laboratories, Bambino Gesu Children’s Hospital, Istituto di Ricovero e Cura a Carattere Scientifico (IRCCS), Rome, Italy

**Keywords:** retinolastoma, extraocular, high-dose chemotherapy, anti-GD2 monoclonal antibodies, intrathecal topotecan

## Abstract

High-dose chemotherapy with autologous stem cell rescue has improved outcomes in patients with metastatic retinoblastoma (RB). However, significant short- and long-term toxicities—especially in very young children with a constitutional *RB1* gene mutation—highlight the need for alternative therapeutic strategies. Monoclonal antibodies targeting tumor-associated antigens such as GD2 have emerged as promising agents in this setting. We report the case of a 2-year-old child diagnosed with extensive left-eye retinoblastoma and massive extraocular dissemination at presentation. The patient was treated with systemic conventional and high-dose chemotherapy combined with intrathecal Topotecan. As consolidation therapy, the child received three courses of the anti-GD2 monoclonal antibody Dinutuximab beta over a 10-day schedule. The patient achieved complete remission and remains disease-free six years after the initial diagnosis. This case suggests that anti-GD2 immunotherapy, used as consolidation treatment, may improve the prognosis of patients with advanced retinoblastoma and potentially reduce the toxicity associated with standard therapies. Further clinical investigation is warranted to validate these findings.

## Introduction

Retinoblastoma (RB) is a highly aggressive tumor and the most common primary intraocular malignancy in children. Metastatic RB is particularly challenging, as it requires intensive, multimodal therapies that often come with substantial toxicities, especially in very young children and even more critically in those with a constitutional *Rb1* mutation. A recent study on patients with metastatic RB treated with high-dose chemotherapy followed by autologous hematopoietic stem cell transplant (HDC-ASCT) reported an overall survival rate grater then 70% for those without CNS metastases. Survival dropped less than 10% for patients with CNS involvement (1, 2). Complications associated with metastatic RB and its treatments include treatment related mortality, hearing loss, neurocognitive deficits and the development of second malignant neoplasms (SMNs), especially in patients carrying genetic predisposition (3–6). This highlights the urgent need for alternative treatment strategies that are both effective and less toxic. In this regard, RB cells, like other neuroectodermal-derived tissues—including neuroblastoma (NB)—highly express GD2, a cell surface disialoganglioside that plays a key role in malignant transformation and is a well-recognized therapeutic target (7, 8). Anti-GD2 monoclonal antibodies (mAbs) have markedly improved outcome in patients with high-risk neuroblastoma (HR-NB) (9–13). Dinutuximab (ch14.18) and Dinutuximab beta (ch14.18/CHO) had been approved by the United States Food and Drug Administration and the European Medicines Agency, respectively, for the treatment of HR-NB. Clinical trials of GD2-targeting therapy are currently ongoing for other GD2-expressing tumors apart from NB (14). According to recent literature, only seven patients with metastatic RB treated with anti-GD2 mAb have been reported (15–17). Anti-GD2 therapy was used as consolidation treatment for minimal residual disease (MRD) following HDC; however, in 2/7 patients, it was administered in the context of active disease. Here, we report a case of metastatic RB, with orbital and multifocal bulky bone disease at diagnosis, managed with anti-GD2 mAb Dinotuximab beta (Db) as consolidation after HDC-ASCT.

## Case presentation

**Figure 1:**
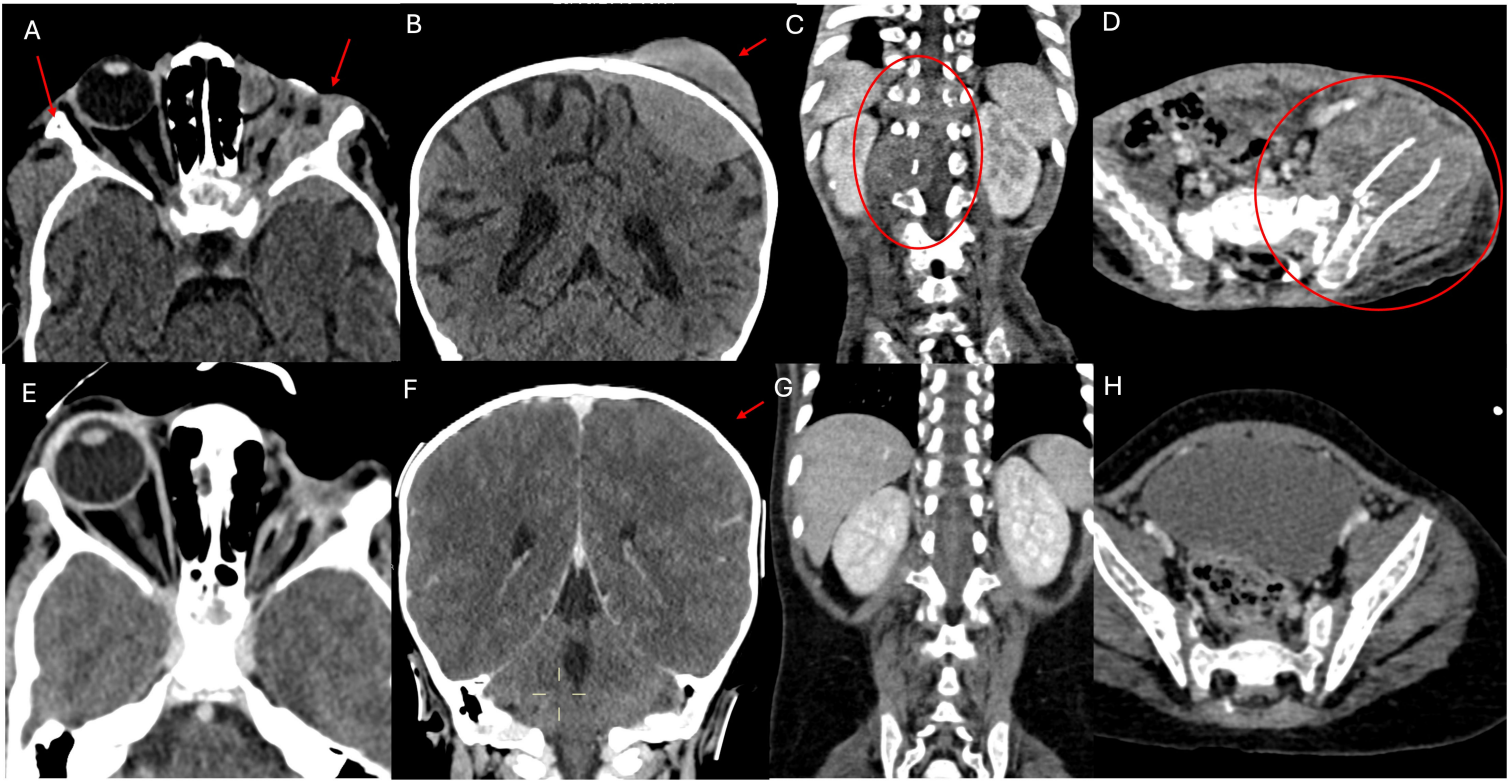
CT scan examination. The first section documents numerous metastatic localizations. **(A)** shows pathological iperdense tissue with high contrast enhancement involving the soft tissues of the left orbit (site of enucleation surgery for RB), infiltrating the local bony structures (greater wings of the sphenoid, frontal and temporal bones), and extending bilaterally to the middle cranial fossa. The left optic nerve is not recognizable, also due to the limited resolution of the CT scan for soft tissues. **(B)** shows an osteodural metastatic localization with high contrast enhancement of the cranial vault on the left side (parietal bone). **(C, D)** respectively show a partially lytic paravertebral mass extending into the spinal canal and a mass centered in the left iliac bone, partially lytic and associated with a fracture, extending to the iliac muscle, both indicative of metastases. **(E–H)** shown the almost complete disappearance of extraorbital metastatic lesions at the end of the treatment.

**Figure 2:**
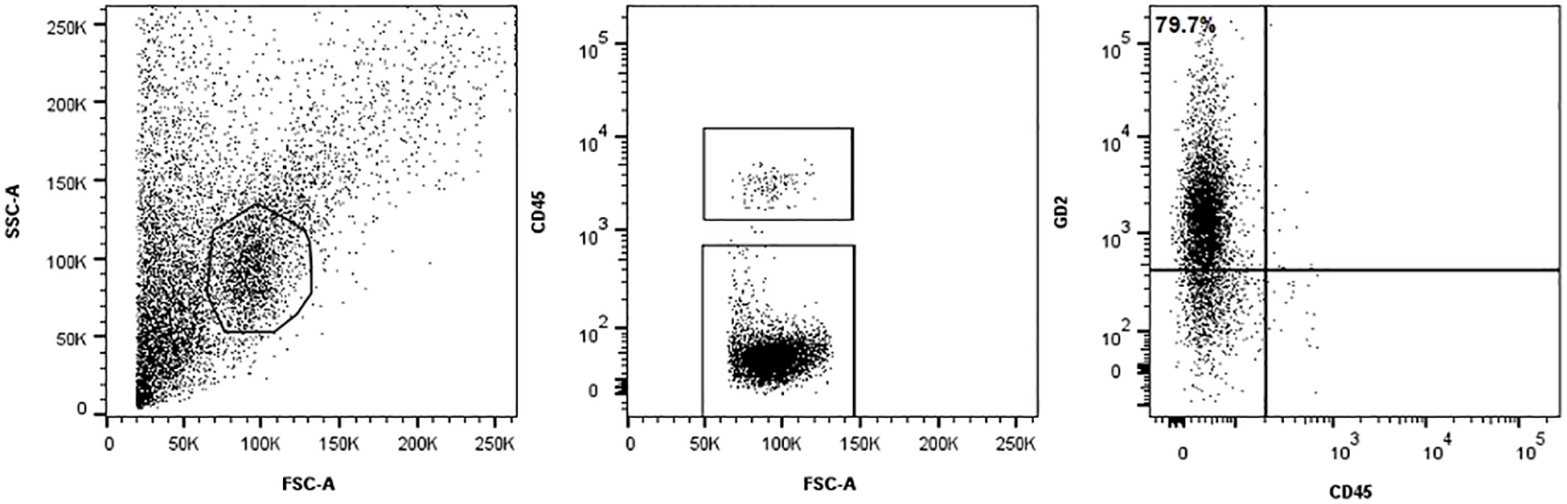
Methods Immunophenotype analysis. The tumor cells were stained with a BV421-conjugated monoclonal mouse anti-human GD2 antibody (clone 14.G2a, BD Biosciences, USA) and anti-human CD45 FITC (BD Biosciences, USA) to identify hematopoietic cells. GD2 expression was evaluated within the CD45-negative single-cell population. Debris was excluded using a morphological gate based on forward and side scatter (FSC/SSC) parameters. To ensure consistency and reliability, isotype controls and peripheral blood mononuclear cells were included as negative controls in flow cytometry run.

A 2-year-old boy was referred to our Hospital with a suspected diagnosis of RB in the left eye. Prior to admission, the patient had undergone orbital exenteration. At the clinical visit, the patient was in poor general condition, with severe malnutrition, generalized bone pain, and limb weakness. A total body CT scan revealed massive extraocular disease extension involving the left orbit and multifocal skeletal lesions (**Figures 1A–D**). A biopsy of a lesion in the right shoulder confirmed the diagnosis of RB. Molecular analysis of the tumor tissue demonstrated a homozygous pathogenic mutation in the *Rb1* gene (c.1959dup). FISH analysis showed a normal copy number of the MYCN gene. The bone marrow biopsy showed infiltration by malignant cells. GD2 expression levels in the CD45-negative cells of the fresh tumor biopsy sample were quantified by flow cytometry and found to be 79.7% (**Figure 2**). Brain and spine MRI showed no evidence of parenchymal or leptomeningeal disease. Cerebrospinal fluid (CSF) analysis was negative for tumor cells. The patient initiated systemic chemotherapy - ICE regimen (Ifosfamide 3gr/mq day 1-2-3, Carboplatin 750mg/mq day 1, Etoposide 150mg/m2 day 1-2-3)-, combined with intrathecal Topotecan (0.4 mg/dose) for CNS prophylaxis. Restaging after four cycles, including whole-body CT and brain and spinal MRI, demonstrated a good partial remission of left orbital and bone lesions. The patient’s general condition progressively improved, including regained mobility and adequate nutritional intake. Constitutional genetic testing for germline *Rb1* alterations was negative. Afterwards the patient underwent HDC according to the COG ARET 0321 protocol (Carboplatin 350mg/mq day 1-2-3/Thiotepa 300mg/mq day 4-5-6/Etoposide 250mg/mq day 4-5-6) followed by ASCT (1). No moderate or severe complications during treatment. Post- HDC whole-body CT imaging showed no radiological evidence of residual disease (**Figures 1E–H**). We did not administer radiotherapy to the sites of bulky disease due to the patient’s young age. Given the high disease burden at diagnosis and the expression of GD2 on tumor cells, anti-GD2 mAb-Db was initiated 60 days after HDC, aiming to replicate the results previously achieved in MRD, following an approach analogous to HR-NB protocols. Three cycles were administered on a compassionate use basis, following informed consent for off-label therapy. The dosing schedule was 100 mg/m² over 10 days, every 5 weeks. Supportive care included morphine and gabapentin. Dinutuximab beta was well tolerated with no serious adverse effects. At the latest follow-up, the patient is in complete remission, with an overall survival of 6 years since diagnosis.

## Discussion

We present the case of a patient diagnosed with metastatic RB who underwent intensive, multimodal therapy. Given the initially inappropriate management and the massive widespread disease, we opted to consolidate remission following myeloablative therapy with Db. This decision was supported by existing clinical experience with GD2-positive tumors, particularly NB. Notably, our patient’s tumor cells, analyzed from a fresh biopsy, showed high GD2 expression—exceeding 75%—which further supported our rationale for using anti-GD2 mAb in this context. Importantly, published studies indicate that optimal response to dinutuximab is observed in cases where more than 75% of tumor cells express GD2, with a threshold of at least 100,000 GD2 molecules per cell considered significant for clinical benefit (18). The multimodal treatment of extraocular RB has significantly improved prognosis, particularly in cases with extra-CNS metastasis. However, notable treatment-related toxicities have been reported. Among the 60 patients enrolled in the COG ARET 0321 trial (1) there were two therapy-related deaths due to septicemia during induction therapy. Additional relevant complications included: encephalopathy in one patient, hearing impairment in four cases, gastric hemorrhage in one. Furthermore, the incidence of SMNs was high: six patients developed second cancers (2/6 received radiotherapy). Radiotherapy is generally reserved for bulky disease not in complete remission after induction chemotherapy. Its use is associated with an increased risk of SMNs within the irradiated field, particularly in patients with an underlying genetic predisposition (3–6). Moreover, due to the typically young age of these patients, radiotherapy is often not recommended. In this context, there is an increasing need for new therapeutic strategies that enhance treatment efficacy while reducing side effects. Dinutuximab is a chimeric monoclonal antibody that targets the disialoganglioside GD2, a glycolipid highly expressed on the surface of several tumor types, including neuroblastoma, small cell lung cancer, and certain sarcomas. By binding to GD2, dinutuximab induces tumor cell lysis through antibody-dependent cell-mediated cytotoxicity (ADCC) and complement-dependent cytotoxicity (CDC). Clinically, it has proven effective primarily in high-risk neuroblastoma, where it is used as maintenance therapy post-consolidation to improve event-free and overall survival. Emerging evidence supports its potential utility in other GD2-expressing malignancies, such as osteosarcoma and triple-negative breast cancer, although these indications are still under active investigation (18, 19). Dinutuximab is administered intravenously, typically as a continuous infusion over 4 consecutive days per treatment cycle, with a standard dose of 17.5 mg/m²/day. Dinutuximab beta, a similar formulation, can be given as a continuous infusion over up to 10 days, which has been associated with reduced pain toxicity compared to shorter infusions. Administration is usually through a central intravenous line to minimize extravasation risk and facilitate management of adverse events. Common side effects include neuropathic pain, fever, hypersensitivity reactions, capillary leak syndrome, and hypotension. Pain management with analgesics, such as opioids and gabapentin, is critical during treatment. Close monitoring for allergic reactions, including anaphylaxis, is mandatory during infusion (20). Recently, seven patients with recurrent extraocular RB treated with anti-GD2 mAb have been reported in the literature (15–17). (**Table 1**) Unlike our case, these patients initially presented with intraocular disease and later developed distant metastases following the failure of conservative treatment. All patients received HDC-ASCT. Patients 1 and 2 were treated with a reduced-intensity myeloablative regimen excluding Carboplatin, due to previous ototoxicity from platinum-based compounds, aiming to preserve hearing in light of their significant visual impairment. Patients 3, 4, 5, and 6 received radiotherapy to sites of bulky disease. Patient 4, developed an alveolar rhabdomyosarcoma of the maxillary bone five years later, located at the periphery of the previous radiation field. The expression of GD2 on tumor sample was assessed only in patients 3, 4, 5, and 6, while for the remaining cases this information was not reported. Patients 3 and 5 had active disease prior to anti-GD2 therapy, allowing for assessment of treatment response: patient 3 showed a complete response of a left mandibular lesion, while patient 5 had a partial response of a left orbital lesion. Our patient also received induction chemotherapy followed by HDC-ASCT, as well as CNS prophylaxis with intrathecal Topotecan. Anti-GD2 therapy was administered during remission after HDC with the intent to eliminate MRD and mitigate the risk of distant relapse, consistent with protocols for HR-NB. Notably, in our patient, the sites of bulky disease identified on imaging after induction chemotherapy were not irradiated, given the patient’s very young age (<3 years). The ability to avoid radiotherapy—along with its well-known long-term toxicities, particularly in very young children—is a significant advantage, highlighting the potential of intensive systemic therapy and targeted immunotherapy to achieve disease control while sparing patients from the harmful effects of radiation. None of the patients reported in the literature, including our case, experienced severe toxicity during anti-GD2 treatment. All adverse effects were effectively managed with appropriate supportive care. Six out of eight patients are alive and in complete remission. Patients 5 and 6 died due to disease relapse in the CNS, occurring 12 and 10 months after anti-GD2 therapy, respectively. This may be attributed to the limited ability of anti-GD2 antibodies to cross the blood-brain barrier, suggesting that CNS disease may require additional therapeutic strategies. These could include cellular immunotherapy employing anti-GD2 Chimeric Antigen Receptor (CAR) T cells (PMID: 37018492) (PMID: 33898999), and/or intrathecal delivery of radiolabeled antibodies, which have already been employed in other solid tumors with CNS metastases (21). All previously reported cases developed distant metastases following the failure of conservative treatments for intraocular tumors. In this context, integrating anti-GD2 mAb into the treatment arsenal for intraocular disease could be a promising strategy. This approach may not only enhance the effectiveness of conservative therapies and improve ocular outcomes, but also help control minimal disseminated disease (MDD) and reduce the risk of extraocular spread. The rationale for such integration is further strengthened by the well-established safety and tolerability profile.

**Table 1:** The characteristics of patients reported in the literature, including our case affected by metastatic RB and treated with anti-GD2 mAb.

Pts	1	2	3	4	5	6	7	8
Age at Dg(m)/Sex	5m/female	12m/female	15m/male	4m/male	10m/male	51m/male	18m/male	24m/male
Initial disease extend/laterality	Intraocular/Bilat	Intraocular/Bilat	Intraocular/Bilat	Intraocular/Bilat	Intraocular/Bilat	Intraocular/Unilat	Intraocular/Bilat	Extraocular/Unilat
Constitutional Rb1 alteration	Yes	Yes	Yes	Yes	Yes	No	Yes	No
Time from Dg to Enucl	Left eye 24m from Dg	Left eye enucl upfront; Right eye 5 yrs from Dg	NR (Unilat Enucl)	NR (Bilat Enucl)	NR (Bilat Enucl)	Enucl upfront	Left eye enucl 24m from Dg; Right eye enucl 43m from Dg	Extenteratio orbitae upfront
Time to extraocular recurrence	7yrs	5 yrs	90m	117m	33m	15m	7,5 yrs	/
Site of Mts	Bone/Liver/CNS MDD	BM, CNS MDD	Bone (paraspinal)/ left mandibula/BM	Rigt temporo-basal dura mater + soft tissue	Left orbit/bone (paraspinal)	Optic nerf/prechiasmatic region	Bone (right tibia)	Left orbit/Bone-multifocal /BM
Therapy for metastatic disease	2cy Ifosfamide/Doxorubicin; 1cy HDC:Thiotepa/Vp16 + ASCT; it Topotecan x 6; Naxitamab/sargramostim	4cy Vcr/Cpm/Vp16; 1cy Thiotepa/Vp16 + ASCT; it Topotecan x 6; Naxitamab/sargramostim	CT according NB 2004-HR; HDC +ASCT; paravertebral mts resection + PT (46 Gy); mandibula PT (50Gy); Db-mAb	CT according NB 2004-HR; HDC +ASCT; CS PT(44,2Gy); Db-mAb	CT according NB 2004-HR; HDC: +ASCT; left orbit PT (50Gy); Db-mAb	CT according NB 2004-HR; HDC: +ASCT; optic nerf PT (45Gy); Db-mAb	4cy Vcr/cisplatin,/cpm/Vp16; HDC Thiotepa/Melphalan +ASCT; Db-mAb	4cy ICE; 1cy Thiotepa/Vp16/Carboplatin + ASCT; it Topotecan x 4; Db-mAb
Cell-surface GD2 antigen expression	NR	NR	Positive	Positive	Positive	Positive	NR	Positive
Time (m) to Anti-GD2 mAb Treatment After ASCT	NR	NR	5m	4m	54 days	5m	NR	2m
Remission Status Prior Anti-GD2 mAb	CR	CR	PR	CR	PR	CR	NR	CR
N and Dose of Anti-GD2 mAb	5cy Naxitamab (3 mg/kg/day, days 1, 3, 5) with sargramostim (500 μg/m²/day, days 0-5)	5cy Naxitamab (3 mg/kg/day, days 1, 3, 5) with sargramostim (500 μg/m²/day, days 0-5)	5cy Db-mAb (100mg/mq over 10 days)	3cy Db-mAb (100mg/mq over 10 days)	5cy Db-mAb (100mg/mq over 10 days)	5cy Db-mAb (100mg/mq over 10 days)	2cy Db-mAb + GM-CSF + IL-2 + spironolactone (doses NR)	3 cy Db-mAb (100mg/mq over 10 days)
Response to Anti-GD2 mAb	NE	NE	Yes/CR	NE	Yes/PR	NE	NE	NE
Outcome	Alive at 10yrs from Dg and 4yrs from Mts	Alive at 8yrs from Dg and 5yrs from Mts	Alive at 45m from start Db-mAb	Alive at 26m from start Db-mAb °	CNS relapse and death 12m and 18m from start Db-mAb	CNS relapse and death 10m and 11m from start Db-mAb	Alive at 18m from start Db-mAb	Alive at 6yrs from Dg
Reference	Larrosa C, et al. (2025) Front. Pediatr (17).	Larrosa C, et al. (2025) Front. Pediatr (17)	Eichholz et al. (2024) Cancer Immunology, Immunoth (15)	Eichholz et al. (2024) Cancer Immunology, Immunoth (15)	Eichholz et al. (2024) Cancer Immunology, Immunoth (15)	Eichholz et al. (2024) Cancer Immunology, Immunoth (15)	Chan et al. (2024) Pediatric Blood & Cancer (16)	This Article

RB, retinobastoma; mAb, monoclonal antibodies; Dg, diagnosis; m, months; Bilat, bilateral; Unilat, unilateral; Enucl, enucleation; Mts, metastasis; ASCT, autologous stem cell transplant; N, number; yrs, years; CNS MDD, central nervous system minimal disseminated disease, BM, bone marrow; cy, cycles; HDC, high dose chemotherapy; it, intrathecal; NR, not reported; NE, not evaluable; CR, complete remission; Vp16, Etoposide; Vcr, Vincristine; Cpm, cyclophosphamide; PT, protontherapy; Gy, Gray; PR, partial remission; Db-mAb; CS, craniospinal; °alveolar Rhabdomyosarcoma right maxilla at 5years from RB Dg (nella discussione inserire la radiot in OS pregressa); ICE, Ifosfamide-Carboplatin-Etoposide.

## Conclusion

Our case is unique compared with those previously reported in the literature due to the extensive disease burden at diagnosis and the successful use of dinutuximab beta as consolidation therapy without the need for radiotherapy. The challenges we encountered included managing a very young patient with severe initial clinical conditions and balancing treatment intensity with long-term toxicity risks. In conclusion, anti-GD2 therapy appears to be a promising option for the control of both MDD and MRD outside the CNS, contributing to a reduced incidence of distant relapse while maintaining a favorable safety profile. This approach may allow for the avoidance of more toxic treatments in very young patients, particularly those with an underlying genetic predisposition. For optimal use of this therapeutic strategy, preliminary assessment of GD2 expression is advisable. Importantly, anti-GD2 mAb should also be considered as a potential component in the management of advanced and/or refractory intraocular disease, with the aim of improving ocular outcomes and minimizing the risk of extraocular dissemination. These encouraging observations strongly support the rationale for designing prospective multinational collaborative trials that incorporate anti-GD2 therapy into the multimodal treatment of retinoblastoma to validate these findings and standardize future therapeutic approaches.

## Data Availability

The data that support the findings of this study are available from the corresponding author upon reasonable request. All data generated or analyzed during this study are included in this published article.
